# Artificial Intelligence in Gastrointestinal Wireless Capsule Endoscopy: A Systematic Literature Review and Meta-Analysis

**DOI:** 10.3390/diagnostics16091269

**Published:** 2026-04-23

**Authors:** Ali Sahafi, Anastasios Koulaouzidis, Amin Naemi

**Affiliations:** 1 Institute of Mechanical and Electrical Engineering, University of Southern Denmark, 5230 Odense, Denmark; alisa@sdu.dk; 2 Department of Clinical Research, University of Southern Denmark, 5000 Odense, Denmark; anastasios.koulaouzidis@rsyd.dk; 3 Department of Biology, University of Southern Denmark, 5230 Odense, Denmark

**Keywords:** systematic literature review, meta-analysis, artificial intelligence, wireless capsule endoscopy

## Abstract

**Background**: Wireless capsule endoscopy is widely used for diagnosing gastrointestinal diseases, but manual interpretation of capsule videos is time-consuming and can vary between clinicians. Artificial intelligence has been increasingly studied to support capsule analysis and reduce clinical workload. This systematic literature review and meta-analysis summarizes current evidence on artificial intelligence methods applied to wireless capsule endoscopy, with a focus on diagnostic performance, validation strategies, and clinical readiness. **Methods**: A systematic search was conducted in PubMed, Scopus, Embase, Web of Science, and Google Scholar. Original journal articles were included based on predefined eligibility criteria. The reviewed studies addressed multiple artificial intelligence tasks, including detection, classification, segmentation, and localization of gastrointestinal abnormalities. **Results**: A total of 72 studies were included. Meta-analysis using random effects models showed high pooled diagnostic performance across clinical indications and gastrointestinal tract locations, with the strongest results reported for bleeding and vascular lesions and more variable performance for inflammatory bowel disease and mixed abnormality categories. The review also identified important clinical and technical barriers that may limit reliability and slow clinical adoption. These included limited external validation, small patient cohorts, retrospective study designs, and inconsistent reporting and evaluation practices. **Conclusions**: Artificial intelligence methods show strong potential to support wireless capsule endoscopy interpretation. Based on the findings, we propose practical recommendations to improve study design and validation. If these recommendations are applied, future studies may report more robust and reliable results, supporting better translation into clinical workflows.

## 1. Introduction

### 1.1. The Global Burden of Gastrointestinal Disorders

The global pattern of Gastrointestinal (GI) diseases is changing rapidly due to a growing wave of cancers, inflammatory conditions, and functional disorders. This trend poses a serious challenge to healthcare systems in many regions. The burden is twofold as developed countries continue to report high rates of GI cancers, while newly industrialized nations are seeing a sharp rise in cases, mainly driven by changes in population structure and lifestyle habits [1,2].

Colorectal Cancer (CRC) and Inflammatory Bowel Disease (IBD) represent the most significant drivers of this expanding burden. CRC remains the third most frequently diagnosed cancer worldwide [2,3], while IBD is rapidly spreading beyond western countries into newly industrialized regions due to urbanization and lifestyle changes [4,5]. Together, these conditions create a critical need for long-term chronic care and regular endoscopic surveillance. This places immense pressure on healthcare systems to improve early diagnosis and match capacity with rising demand.

Digestive diseases also impose a major clinical and financial burden on healthcare systems. In 2019, around 332 million people in Europe were living with digestive conditions, which resulted in 498,000 GI-related deaths and over €20 billion in inpatient care costs annually along with significant indirect losses due to reduced productivity [6]. The rising demand to investigate symptoms like Obscure Gastrointestinal Bleeding (OGIB), iron-deficiency anemia, and chronic abdominal pain has added further pressure on endoscopy procedures [7].

### 1.2. Wireless Capsule Endoscopy

**Figure 1:**
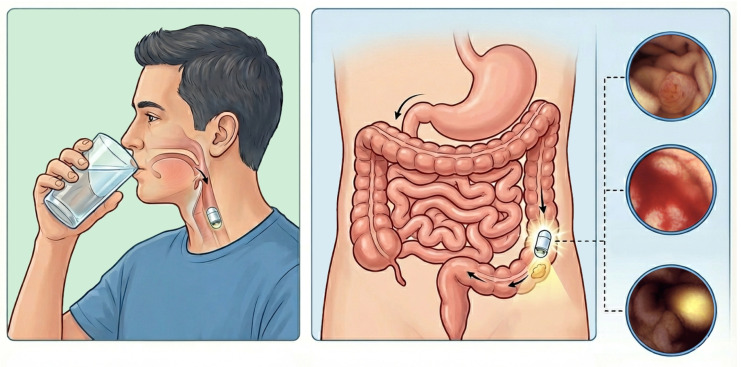
WCE detecting GI disorders.

Wireless Capsule Endoscopy (WCE), introduced in 2000, is a breakthrough imaging technology that has transformed the diagnosis of GI disorders [8]. Unlike conventional endoscopy or radiology, WCE allows for direct visualization of the entire GI tract using a swallowable capsule with a tiny camera, light source, battery, and wireless transmitter (Figure 1). Over the past two decades, WCE has become a core tool in gastroenterology and is now widely used as a first-line method for small bowel investigation. Ongoing advancements in both hardware and software have significantly improved its imaging performance [9]. Modern capsules now include higher-resolution sensors, wider fields of view, and extended battery life, typically operating 8 to 12 h and capturing 50,000 to 60,000 images per exam [10]. Newer models also offer features such as adaptive frame rates, enhanced optics, and broader viewing angles, which contribute to better image quality and more reliable lesion detection. In addition, specialized capsule designs have been introduced for the esophagus, colon, and stomach, expanding the diagnostic reach of WCE [11].

From a clinical perspective, WCE has significantly improved the detection and assessment of GI diseases. Its primary indication is the investigation of OGIB and unexplained iron deficiency anemia. In these cases, WCE enables full visualization of the small bowel mucosa and often reveals underlying lesions such as angioectasias, ulcers, or tumors that were missed by conventional methods [8]. WCE is also a valuable tool in the diagnosis of Crohn’s and Ulcerative Colitis (UC), as it can detect inflammatory lesions such as aphthous ulcers or erosions in the small bowel that are beyond the reach of ileocolonoscopy. This is especially useful for patients with suggestive symptoms but normal findings on standard endoscopy. Additionally, WCE plays an important role in detecting small bowel tumors and in surveillance of polyposis syndromes like Peutz–Jeghers syndrome by offering a noninvasive method to examine the entire intestine for polyps or neoplastic lesions [12]. It is also a convenient alternative for patients who are unable to undergo sedation or who have experienced an incomplete colonoscopy [8]. Notably, recent developments are shifting the field toward pan-enteric capsules that image both the small bowel and colon in a single examination. This supports a broader move toward whole-gut assessment.

### 1.3. The Limitations of Human Interpretation

WCE generates a high-volume video stream consisting of tens of thousands of frames over several hours that requires manual review. Interpreting a single examination typically takes around 45 min of physician time [13]. Since only a small fraction of frames contain relevant pathology, it is not uncommon for clinicians to overlook subtle lesions or misclassify normal findings. Retrospective studies have reported notable miss rates as human readers failed to detect 5.9% of vascular lesions, 0.5% of ulcers, and up to 18.9% of neoplastic lesions on WCE [14]. These omissions often result from the difficulty of identifying small or subtle abnormalities within largely normal mucosa, as well as from considerable inter-observer variability. For example, a study by the I-CARE Group [15] reported that only 23% of inter-reader comparisons achieved good or near-perfect agreement, which highlights inconsistencies across different reviewers. Diagnostic accuracy is further diminished by fatigue, which can reduce performance after reviewing even one full-length recording [15]. These human limitations underscore the need for assistive technologies that can enhance both efficiency and consistency in WCE interpretation.

### 1.4. The Promise of Artificial Intelligence

Artificial Intelligence (AI), particularly deep learning, has emerged as a promising approach to overcome the challenges associated with WCE interpretation. AI-based systems can operate as a second reader or even perform an initial screening of WCE data to reduce workload and improve diagnostic accuracy. Several AI-assisted platforms are now capable of automatically detecting frames with potential lesions while skipping those without relevant findings, significantly reducing the manual review burden [16]. For example, proprietary capsule software modes such as QuickView or ExpressView use algorithms to highlight suspected pathology and filter out normal frames. In practice, these tools have reduced the number of frames requiring clinician review by up to 20-fold [13].

Convolutional Neural Networks (CNNs) are among the first successful applications that enable automated detection of common abnormalities such as ulcers, angioectasias, polyps, and bleeding with high accuracy [17,18,19,20,21]. CNN-based algorithms consistently achieve classification accuracies above 90%, with pooled sensitivities between 95% and 98% reported for ulcer and bleeding detection [17]. This level of performance has been reported to be comparable to expert endoscopists in specific studies and may represent an important advance in reducing diagnostic errors. In addition to classification, more advanced computer vision models have been used to localize lesions within individual frames. Single-shot object detectors such as You Only Look Once (YOLO) networks [22] and Single Shot MultiBox Detector (SSD) [23] have been adapted for WCE images to draw bounding boxes around abnormalities and mark their precise locations. These models support real-time detection of lesions such as polyps or bleeding during video playback.

The latest generation of deep learning models, Vision Transformers (ViTs), has shown strong potential in WCE applications. Unlike CNNs, which extract localized features through convolutional filters, ViTs use self-attention mechanisms to capture long-range dependencies and global context across the entire image. This ability to model full-frame relationships offers a distinct advantage in WCE, where lesions often appear subtle, dispersed, or embedded within a broader mucosal background. Recent studies have demonstrated that ViT-based architectures can match or surpass CNNs in detecting GI lesions such as bleeding, ulcers, and polyps [24]. ViTs also show improved robustness to image variability and stronger generalization across datasets and imaging conditions. These strengths make ViTs well-suited for the large-scale, visually complex image streams generated by WCE [24].

In summary, AI methods achieve high performance in detecting GI lesions on WCE. In research studies, their performance often matches or surpasses expert readers. These systems can identify subtle abnormalities that may go unnoticed in manual review and reduce the time required for interpretation.

## 2. Research Gaps and Objectives

The contributions of this study are threefold:

(1) Almost all review articles on the use of AI in WCE applications are restricted to specific settings, such as particular diseases, GI locations, or predefined applications. To address this limitation, in the current study we extracted all original journal research articles that applied AI for the analysis of WCE outputs. In other words, our search strategy was not restricted to specific diseases, applications, or GI locations.
(2) Moreover, almost all studies in this domain have largely focused on analyzing and comparing the performance metric values. However, several critical technical and clinical barriers must be considered to improve the trustworthiness of AI results and to increase the likelihood of successful deployment of AI systems in clinical practice. Therefore, in this study, we go beyond performance metrics and highlight these barriers and provide recommendations for future research.
(3) Finally, this study presents a more comprehensive meta-analysis by systematically structuring the results across two complementary dimensions including clinical indication–based domains and GI tract locations. The included studies were first grouped into five categories based on their clinical indications, and subsequently reclassified into four categories according to the GI tract locations.

## 3. Method

This systematic review was conducted in accordance with the Preferred Reporting Items for Systematic Reviews and Meta-Analyses (PRISMA) [25] guidelines and focused on the questions listed in Table 1.

### 3.1. Search Strategy and Study Selection

We searched four databases, including PubMed, Scopus, Embase (Ovid), and Web of Science. We also searched for articles on Google Scholar. To find relevant studies, we used a mixure of keywords and filters organized into five categories as AI terms, technical terms, medical terms, document type, publication year, and language. Keywords within each category were connected using OR, while the categories were combined using AND. Table 2 shows the search criteria of this study. The full queries for databases are presented in Supplementary File S1.

We considered different criteria to include or exclude of studies. These conditions are presented in Table 3.

### 3.2. Data Extraction

Two researchers (AN, AS) individually examined the titles and abstracts using the Covidence tool. Subsequently, they conducted a full-text review, resolving disagreements with a senior researcher (AK), who decided on the inclusion or exclusion of the article. The extracted data was compiled into spreadsheets using the Critical Appraisal and Data Extraction for Systematic Reviews (CHARMS) checklist [26].

### 3.3. Risk of Bias Assessment

Risk of Bias (ROB) assessment of each study was investigated using Quality Assessment of Diagnostic Accuracy Studies 2 (QUADAS-2) tool [27]. It evaluates four key domains: *Patient Selection*, *Index Test*, *Reference Standard*, and *Flow and Timing*. QUADAS-2 ensures the reliability and generalizability of study findings by assigning three levels of bias as *Low*, *Unclear*, and *High* to each domain for each study.

### 3.4. Meta-Analysis

In this study, we conducted a meta-analysis using a random-effects model to synthesize findings across multiple studies while accounting for heterogeneity [28]. We assessed heterogeneity using Cochran’s Q, I^2^, and $\tau^{2}$, ensuring that our pooled results reflect study variability and enhance the robustness of our conclusions. Four commonly reported performance metrics, including accuracy, sensitivity, specificity, and Area Under the Curve (AUC) were considered to quantitatively synthesize the diagnostic performance of AI models across studies.

To perform the meta-analysis, two complementary stratification strategies were considered. First, a clinical indication-based approach, which groups studies according to the targeted GI pathology, was implemented. Then, a GI tract location-based approach, which categorizes studies according to the anatomical region of the GI tract, was investigated.

In the first scenario, studies were categorized into five clinically meaningful groups based on the primary GI pathology. *IBD (Crohn’s disease, UC)* included studies focusing on IBD activity, severity assessment, or disease-related lesions. *Polyp, Tumor, Protruded Lesion* comprised studies targeting protruding mucosal abnormalities. *Ulcer, Erosion, Inflammation* included studies detecting mucosal breaks and inflammatory lesions not specific to IBD. *Bleeding, Vascular* covered studies addressing GI bleeding and vascular abnormalities. *Mixed, General Abnormality* referred to studies detecting multiple lesion types or general abnormalities without a single dominant focus.

To ensure consistency across studies with different reporting styles, outcome definitions were harmonized by grouping similar lesion types into these broader categories based on their clinical and visual similarity in capsule endoscopy. This approach reduced variability in outcome definitions and allowed more reliable pooling of performance metrics using random-effects models.

In the second scenario, studies were categorized based on the anatomical region of the GI tract. Four main groups were defined. The *Small Bowel* category included studies focusing on small bowel capsule endoscopy. The *Colon* category included studies targeting colonic evaluation. The *Entire GI Tract* category referred to studies analyzing the full GI tract without restriction to a specific region. The *Different Parts of GI Tract* category included studies that evaluated multiple anatomical regions separately or in combination. This categorization allowed comparison of AI performance across different GI locations while considering differences in visual features and disease distribution.

## 4. Results

**Figure 2:**
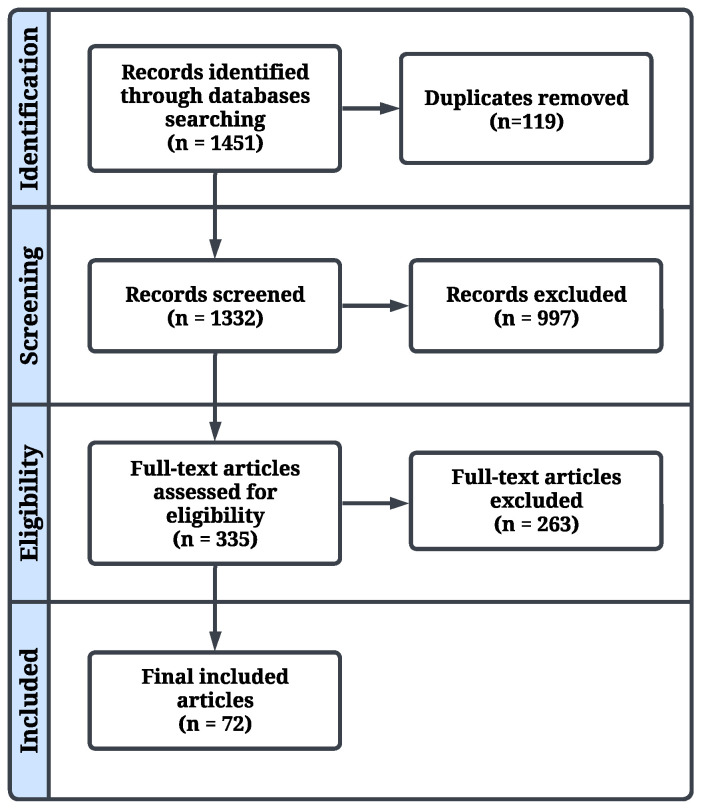
PRISMA diagram.

The initial search across all databases yielded 1451 records after automatic duplicate removal using Covidence. During subsequent manual screening, 119 additional duplicate records were identified that had not been detected by Covidence and were therefore removed. After eligibility screening, full-text assessment, quality appraisal, and reference checking of included studies, a total of 72 studies were finally included. The PRISMA chart of the study selection process is illustrated in Figure 2. The PRISMA checklist is also presented as Supplementary File S2.

The extracted data from final set of studies is presented in two tables in the Appendix A as Table A1 and Table A2. Table A1 summarizes the general characteristics of the studies, while Table A2 presents the AI models and performance metrics reported in the included studies. The complete CHARMS data extraction spreadsheet is also provided as Supplementary File S3.

### 4.1. Geographical Distribution

**Figure 3:**
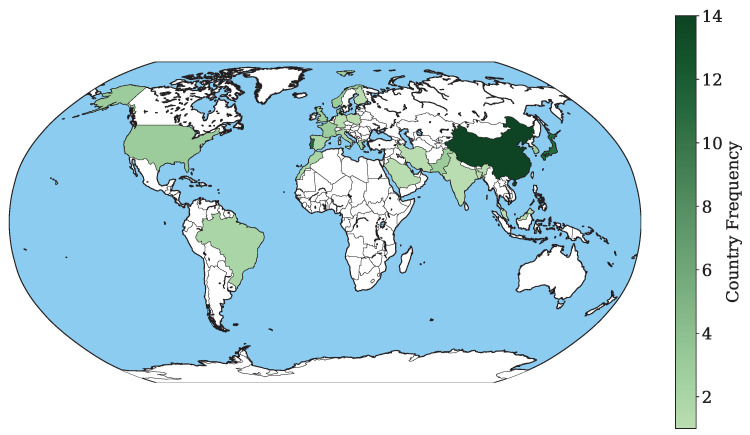
Geographical distribution of included studies.

Figure 3, derived from Table A1, visualizes the international distribution of studies on AI applications in WCE. As seen, research output is heavily concentrated in East Asia and Europe. China shows the highest research output (n = 14), followed by Portugal (n = 12) and Japan (n = 11). Other countries with notable research activity include South Korea (n = 6), Denmark (n = 5), and Israel (n = 5). Additional research activity is observed in several European and North American countries, including the UK (n = 4), Spain (n = 4), France (n = 4), and the USA (n = 4). Further studies are published from Pakistan and Norway (each n = 3).

### 4.2. Publication Trend

**Figure 4:**
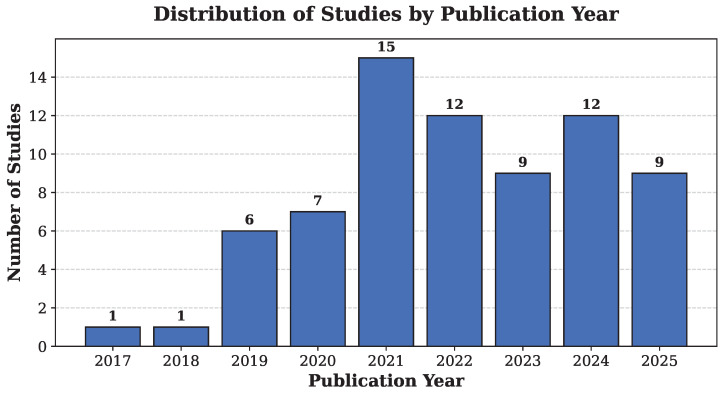
Publication trend of included studies.

Figure 4, extracted from Table A1, shows the distribution of included studies by publication year. The number of studies increased steadily after 2019, with a clear rise from 2020 onward and a peak in 2021. Although minor fluctuations are observed in subsequent years, the overall trend indicates growing research interest in AI applications for capsule endoscopy in recent years.

### 4.3. Cohort Presentation

**Figure 5:**
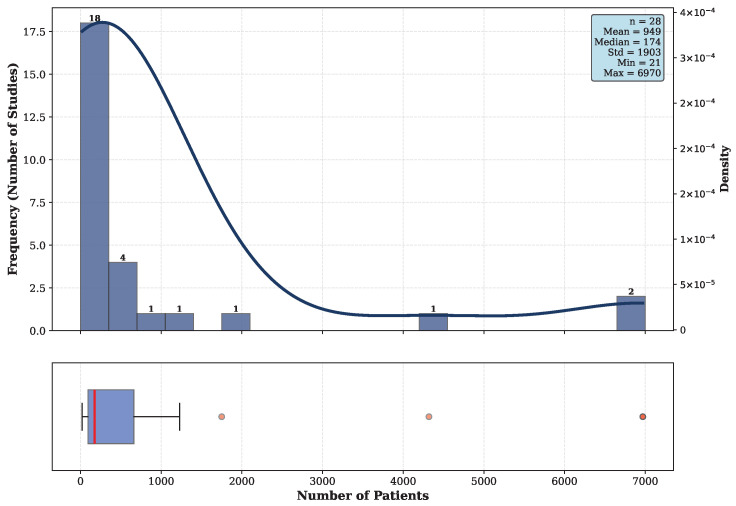
Distribution of number of patients across included studies.

The number of patients included in each study is one of the important cohort-related aspect. This parameter can indirectly reflect the robustness of the findings [29]. Figure 5, extracted from Table A1, presents the distribution of patient numbers for 28 studies that reported this information. The upper panel shows a histogram with an overlaid Kernel Density Estimation (KDE) curve. The lower panel highlights substantial heterogeneity in cohort sizes, ranging from 21 to 6970 patients. The median and mean numbers of patients across the included studies were 174 and 949, respectively. However, the majority of studies (53.6%) included fewer than 400 patients, indicating that most investigations in this field are small to moderate in size.

**Figure 6:**
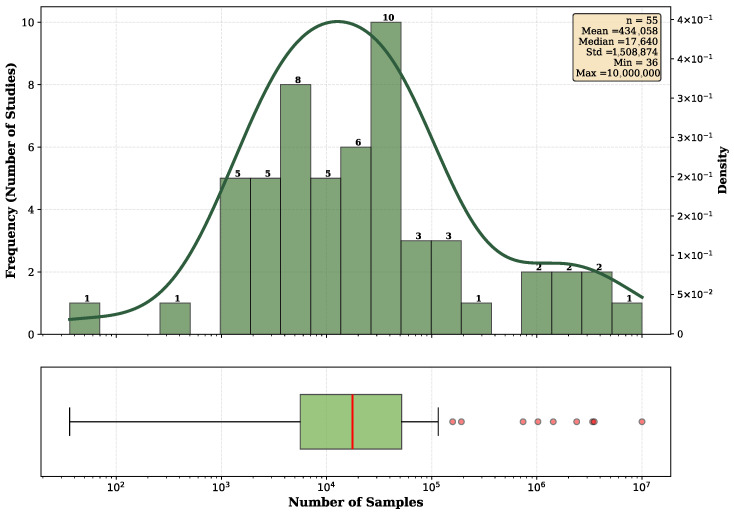
Distribution of the number of patients across included studies.

In addition to the number of patients, the total number of samples in each dataset is an important indicator of the reliability of AI results. Sample size information was available for 55 studies, and Figure 6 shows the distribution of sample sizes. The upper panel displays a histogram with logarithmic x-axis scaling and an overlaid KDE curve illustrating the distribution of sample sizes. The lower panel presents a box plot with logarithmic scaling that reveals extreme variability spanning seven orders of magnitude, from 36 to 10,000,000 samples. The median and mean numbers of samples across the included studies were 17,640 and 434,058, respectively. The distribution shows a substantial right skew, driven by a small number of large-scale studies using extensive retrospective databases, while most studies (over 70%) analyzed fewer than 50,000 capsule endoscopy samples.

### 4.4. GI Tract Locations

**Figure 7:**
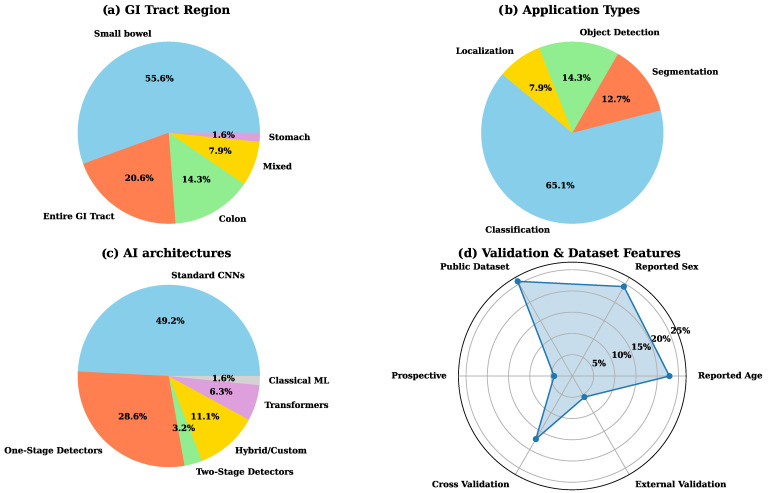
Distribution of included studies based on (**a**) GI tract location, (**b**) Application type, (**c**) AI architecture, (**d**) Validation strategy and dataset features.

Based on Table A1 and as shown in Figure 7a, the majority of included studies focused on the small bowel (55.6%), followed by entire GI tract (20.6%), colon (14.3%), mixed regions (7.9%), and stomach (1.6%). This distribution indicates a notable emphasis on small bowel research compared to other regions of the GI tract.

### 4.5. Applications

AI models have been applied to WCE across four primary tasks, including classification, segmentation, object detection, and localization (Figure 7b and Table A2). Among these, classification is the most widely explored application, accounting for 65.1% of studies. In classification tasks, a key application is the automatic filtering of abnormal images from the large number of frames generated during WCE procedures. Since a single examination can produce tens of thousands of images, AI-based methods help reduce the clinician’s workload by identifying frames that contain potential abnormalities such as bleeding, ulcers, and polyps.

Segmentation accounts for 12.7% of the included studies and provides pixel-level delineation of anatomical structures and lesions. This supports quantitative assessment such as lesion area and boundaries. It can aid severity grading and treatment planning. Object detection, reported in 14.3% of studies, localizes abnormalities using bounding boxes. Compared with segmentation, it is less precise but faster and often sufficient for rapid screening and triage of suspicious frames.

Finally, localization, which accounts for 7.9% of the studies, focuses on mapping detected abnormalities to their corresponding anatomical locations within the GI tract. This application is particularly important for guiding further diagnostic and therapeutic interventions, as it enables precise targeting of lesions identified by WCE during follow-up endoscopic procedures.

### 4.6. AI Models

Based on Table A2 and Figure 7c, standard CNNs, such as ResNet, VGG, DenseNet, Xception, and Inception, were the most frequently used models, accounting for approximately 49% of the included studies. Many of these CNN-based methods incorporate transfer learning, feature-fusion layers, or customized modules.

Object detection frameworks accounted for approximately 32% of the included studies, combining both one-stage and two-stage detectors. Within this group, single-pass methods such as YOLO (v3, v5, v8) and SSD were commonly used because of their ability to localize lesions, including polyps and angiodysplasias, with high speed and accuracy.

Transformer-based models, including ViTs and TimeSformer, were used in approximately 6% of the studies. It indicates growing interest in self-attention mechanisms for modeling the spatial and temporal characteristics of WCE data. Hybrid or custom architectures, such as Capsule Networks (CapsNet), Symmetric Positive Definite Network (SPDNet), and proprietary ensemble models, accounted for about 11% of the included studies. These models often incorporated specialized attention mechanisms or domain-adversarial training to address challenges such as data imbalance and domain shift. In contrast, only one study employed a classical machine learning algorithm.

### 4.7. Study Design and Validation Strategies

As seen in Table A1, most studies have not provided information about their cohort. The main information about the patient population in some studies is age and sex statistics. These variables are primarily presented as mean and median values, although a significant proportion (approximately 75%) did not provide age data explicitly (Figure 7d).

The gender column reflects the percentage of male or female participants. The percentage of males in the included studies ranged from approximately 45% to 73%. However, a large portion of the studies (approximately 75%) did not explicitly state the male or female proportion (Figure 7d). Studies with reported male dominance often cited percentages exceeding 60% that highlights a trend toward a predominantly male sample in most datasets [30,31,32,33,34].

As seen in Table A2, the included studies utilized a variety of performance metrics, including sensitivity, specificity, accuracy, precision, Area Under the Curve (AUC), recall, Positive Predictive Value (PPV), Negative Predictive Value (NPV), and Matthews Correlation Coefficient (MCC) to evaluate their AI models. Among these, sensitivity, specificity, accuracy, and AUC were the most commonly reported metrics.

Validation and study design practices are summarized in Figure 7d. Only about 20% of studies explicitly reported the use of cross-validation, while external validation was reported in just 7% of studies. In addition, approximately 25% of studies relied on public datasets, and only a small proportion (about 5%) employed a prospective study design. The limited use of cross-validation, external validation, and prospective designs raises concerns about model robustness and generalizability across diverse clinical settings.

### 4.8. ROB Assessment

**Figure 8:**
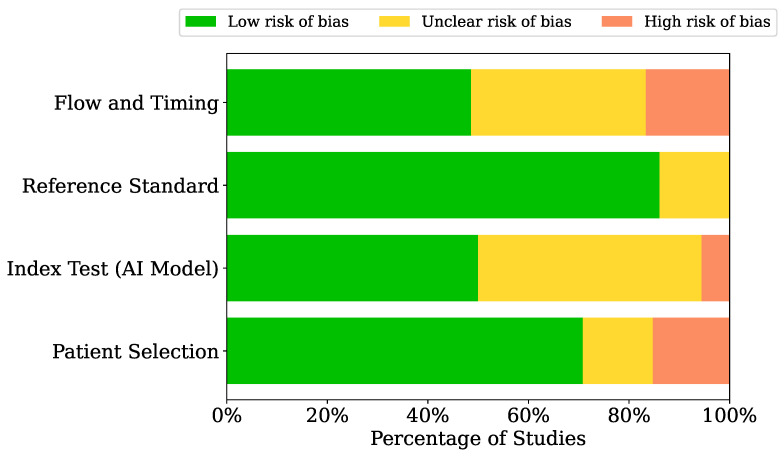
ROB analysis of included studies using the QUADAS-2 tool.

Figure 8 presents the summary of ROB analysis. The complete ROB assessment for each included study is provided in Supplementary File S4. The results show that *Patient Selection*, *Reference Standard*, and *Flow and Timing* domains have a low ROB, with most studies falling into this category. However, the *Index Test (AI Model)* domain shows a mix of low and unclear ROB, which indicates that, despite some studies following rigorous methods, others had unclear or poorly reported details regarding the AI model’s performance.

### 4.9. Meta-Analysis

**Figure 9:**
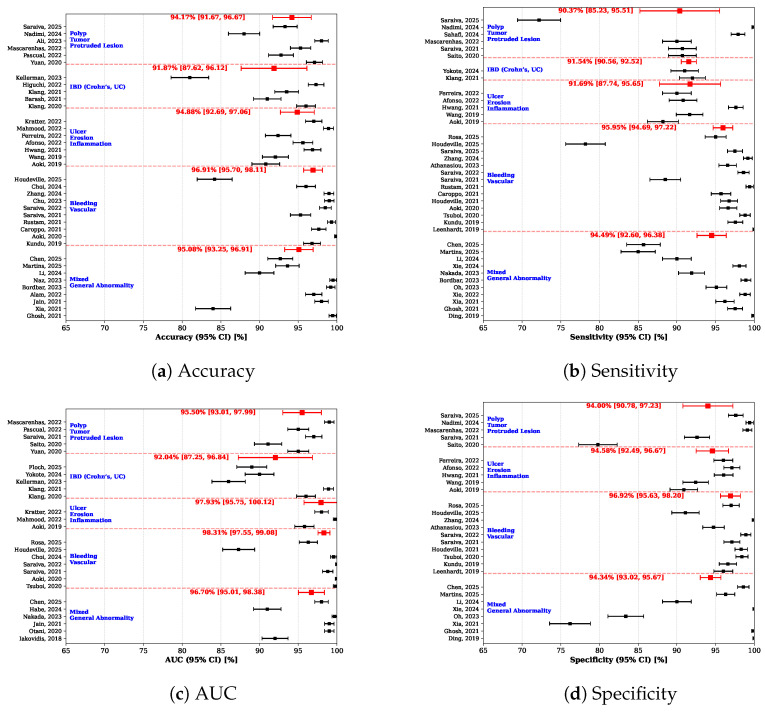
Results of meta-analysis by clinical indication: (**a**) Accuracy, (**b**) Sensitivity, (**c**) AUC, and (**d**) Specificity. Included studies: Saraiva et al. [35], Nadimi et al. [36], Ali et al. [37], Mascarenhas et al. [38], Pascual et al. [39], Yuan et al. [40], Kellerman et al. [41], Higuchi et al. [42], Klang et al. [43], Barash et al. [44], Klang et al. [45], Kratter et al. [46], Mahmood et al. [47], Ferreira et al. [48], Afonso et al. [49], Hwang et al. [50], Wang et al. [31], Aoki et al. [51], Houdeville et al. [52], Choi et al. [53], Zhang et al. [54], Chu et al. [55], Saraiva et al. [56], Saraiva et al. [57], Rustam et al. [58], Caroppo et al. [59], Aoki et al. [60], Kundu et al. [61], Chen et al. [62], Martins et al. [63], Li et al. [64], Naz et al. [65], Bordbar et al. [34], Alam et al. [66], Jain et al. [67], Xia et al. [68], Ghosh et al. [69], Sahafi et al. [22], Saito et al. [32], Yokote et al. [70], Rosa et al. [71], Athanasiou et al. [72], Tsuboi et al. [73], Leenhardt et al. [30], Xie et al. [33], Nakada et al. [74], Oh et al. [75], Xie et al. [76], Ding et al. [77], Floch et al. [78], Kellerman et al. [41], Otani et al. [79], Iakovidis et al. [80], Habe et al. [81], Houdeville et al. [82].

The results of the clinical indication–based scenario are presented in Figure 9. The statistics for this approach is provided as Supplementary File S5.

Across all categories, the pooled estimates were consistently high, indicating strong diagnostic performance of AI-based systems in WCE. The highest pooled accuracy and AUC values were observed in the *Bleeding, Vascular* category (accuracy = 96.91%; AUC = 98.31%), that reflect the visually distinctive nature of bleeding-related findings and their suitability for automated detection. Similarly, studies focusing on *Ulcer, Erosion, Inflammation* (accuracy = 94.88%, AUC = 97.93%) and *Polyp, Tumor, Protruded Lesion* (accuracy = 94.17%; AUC = 95.50%) demonstrated robust pooled performance across all metrics, with narrow confidence intervals, which suggests reliable and reproducible results despite inter-study heterogeneity.

In contrast, the *IBD (Crohn’s, UC)* category showed slightly lower pooled estimates, particularly for AUC (92.04%), with wider confidence intervals for some metrics like AUC and specificity. Similarly, the *Mixed, General Abnormality* category showed moderate pooled estimates with sensitivity and specificity (sensitivity = 94.49%, specificity = 94.34%). This may reflect the greater phenotypic variability and diagnostic complexity of these conditions, as well as differences in annotation strategies and reference standards across studies. Nevertheless, the overall high pooled performance across all categories supports the effectiveness of AI approaches for diverse clinical applications in capsule endoscopy.

**Figure 10:**
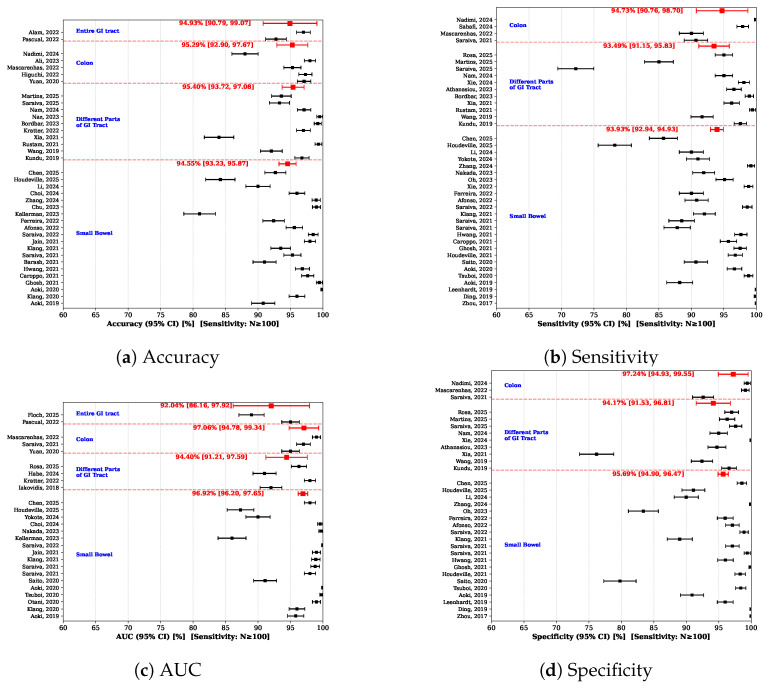
Results of meta-analysis by GI location: (**a**) Accuracy, (**b**) Sensitivity, (**c**) AUC, and (**d**) Specificity. Included studies in order of appearance: Alam et al. [66], Pascual et al. [39], Nadimi et al. [36], Ali et al. [37], Mascarenhas et al. [38], Higuchi et al. [42], Yuan et al. [40], Martins et al. [63], Saraiva et al. [35], Nam et al. [83], Naz et al. [65], Bordbar et al. [34], Kratter et al. [46], Xia et al. [68], Rustam et al. [58], Wang et al. [31], Kundu et al. [61], Chen et al. [62], Houdeville et al. [52], Li et al. [64], Choi et al. [53], Zhang et al. [54], Chu et al. [55], Kellerman et al. [41], Ferreira et al. [48], Afonso et al. [49], Saraiva et al. [56], Jain et al. [67], Klang et al. [43], Saraiva et al. [57], Barash et al. [44], Hwang et al. [50], Caroppo et al. [59], Ghosh et al. [69], Aoki et al. [60], Klang et al. [45], Aoki et al. [51], Sahafi et al. [22], Saravia et al. [84], Rosa et al. [71], Xie et al. [33], Athanasiou et al. [72], Yokote et al. [70], Nakada et al. [74], Oh et al. [75], Saravia et al. [56], Saravia et al. [57], Saravia et al. [85], Tsuboi et al. [73], Saito et al. [32], Aoki et al. [51], Leenhardt et al. [30], Xie et al. [76], Ding et al. [77], Iakovidis et al. [80], Otani et al. [79], Zhou et al. [86], Habe et al. [81], Houdeville et al. [82].

Similarly, the statistics for the second scenario is provided as Supplementary File S6. As seen in Figure 10, across all GI regions, the pooled estimates are consistently high, indicating strong diagnostic performance of AI-based methods in capsule endoscopy. Studies focusing on the *Small Bowel* formed the largest subgroup and demonstrated stable pooled values across all metrics (accuracy = 94.55%, sensitivity = 93.93%, AUC = 96.92%, specificity = 95.69%), which reflects the advanced development and widespread clinical use of capsule endoscopy in this region.

Studies analyzing the *Entire GI tract* (accuracy = 94.93%, AUC = 92.04%), *Colon* (accuracy = 95.29%, sensitivity = 94.73%, specificity = 97.24%, AUC = 97.06%), and *Different Parts of GI Tract* (accuracy = 95.40%, sensitivity = 93.49%, specificity = 94.17%, AUC = 94.40%) also showed strong pooled performance, although with slightly wider confidence intervals in some metrics, likely due to increased methodological and clinical heterogeneity. Overall, these results suggest that AI models achieve reliable and generalizable performance across different GI locations, while regional characteristics may still contribute to variability in diagnostic outcomes.

**Figure 11:**
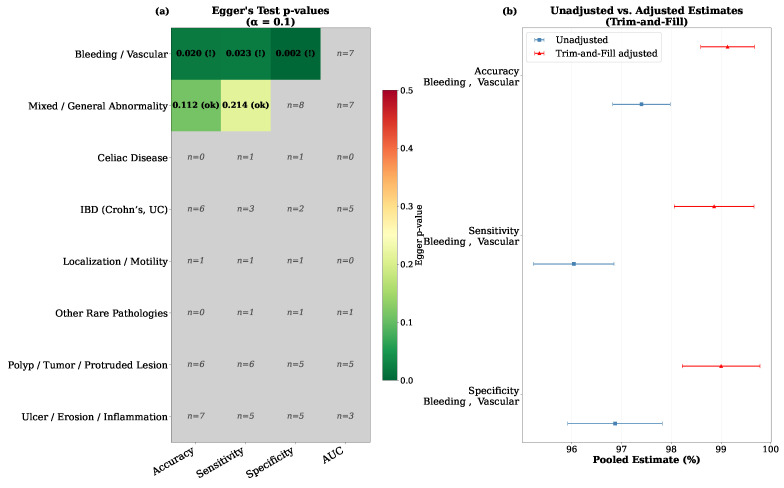
(**a**) Summary of publication bias assessment across all subgroups, and (**b**) Comparison of unadjusted versus trim-and-fill-adjusted pooled estimates.

Publication bias was assessed using Egger’s linear regression test (α=0.10) [87] and the Duval & Tweedie trim-and-fill method [88] for all metric–subgroup combinations with at least 10 studies. A summary heatmap of Egger’s test p-values across all subgroups is presented in Figure 11, where colour intensity reflects the degree of asymmetry. Subgroups with fewer than 10 studies were not formally tested and are shown as grey cells. Also, cells labelled as (!) for significant asymmetry (p<0.10) and (ok) for no significant asymmetry. Subgroups with fewer than 10 studies are shown as grey cells annotated with their study count and were excluded from formal testing.

**Figure 12:**
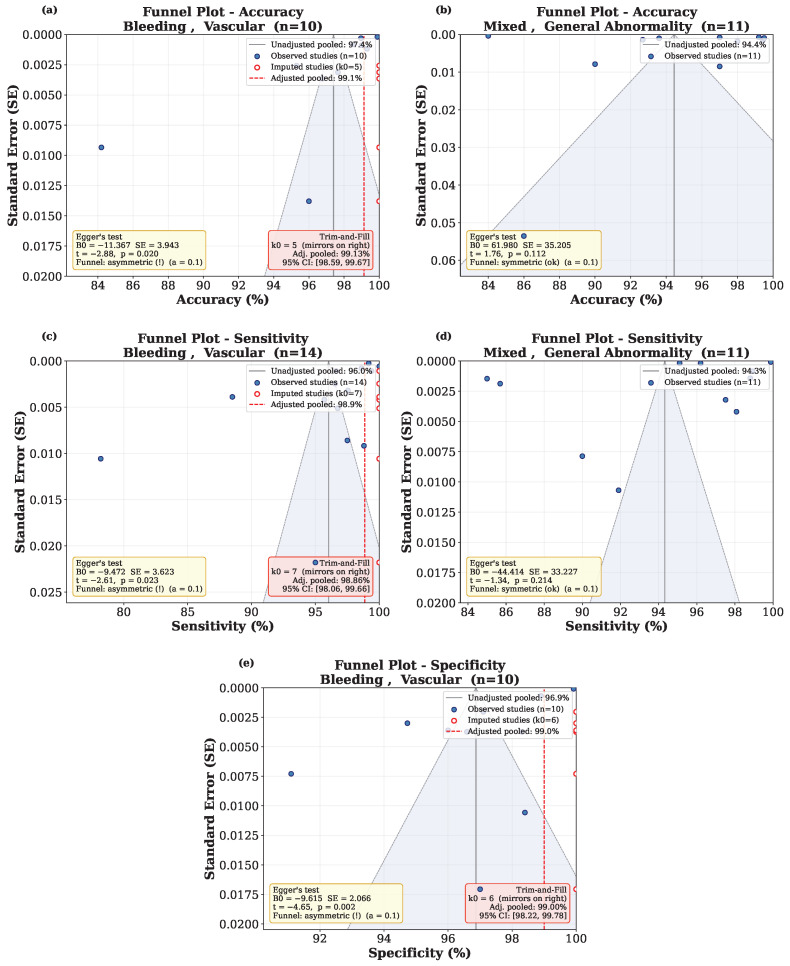
Funnel plots for subgroups with n≥10, (**a**) Accuracy for *Bleeding, Vascular*, (**b**) Accuracy for *Mixed, General Abnormality*, (**c**) Sensitivity for *Bleeding, Vascular*, (**d**) Sensitivity for *Mixed, General Abnormality*, (**e**) Specificity for *Bleeding, Vascular*.

Funnel plots were constructed by plotting study-level estimates against the inverted standard error, with the 95% pseudo-confidence interval shaded and the DerSimonian-Laird pooled estimate indicated by a solid vertical line (Figure 12). Five combinations met the inclusion criteria, including accuracy, sensitivity, and specificity within the *Bleeding, Vascular* category, and accuracy and sensitivity within *Mixed, General Abnormality*. As seen in Figure 12, significant funnel plot asymmetry was observed in the *Bleeding, Vascular* subgroup for all three metrics (accuracy p=0.020, sensitivity p=0.023, specificity p=0.002), whereas no significant asymmetry was detected in *Mixed, General Abnormality* (accuracy p=0.112, sensitivity p=0.214), as also illustrated in the funnel plots. Notably, the observed asymmetry was in a conservative direction, with smaller studies reporting lower performance than larger studies. The Duval & Tweedie trim-and-fill method imputed additional studies on the higher-performance side that results in slightly increased adjusted estimates compared with the original values. The adjusted estimates were 99.13% versus 97.40% for accuracy, 98.86% versus 96.04% for sensitivity, and 99% versus 96.87% for specificity. These results indicate that the pooled estimates are unlikely to be inflated by publication bias. It is also worth noting that due to the high diagnostic performance observed (>95% in most subgroups), the funnel plot analysis is subject to a ceiling effect.

## 5. Discussion

This systematic review highlights the strong diagnostic performance of AI models in capsule endoscopy while uncovering critical limitations in validation practices, dataset diversity, and clinical readiness.

Many prior reviews have constrained their scope to narrow disease categories such as celiac disease [89] or IBD [90], excluding broader AI applications. Others focus exclusively on specific lesion types like protruded lesions or bleeding sources [17,91,92,93,94]. However, in this study, we considered broader domain to cover more AI-related studies on the WCE applications.

Although many studies have investigated the use of AI across different WCE applications, challenges related to real-world deployment remain largely underexplored. In of the Discussion, we address the limitations of the current research landscape and outline the technical and clinical barriers that must be addressed in future studies to achieve robust and reliable AI results and to support effective integration into real-world clinical practice.

### 5.1. Datasets

First, one of the main factors affecting the quality and trustworthiness of AI models is the quality and diversity of the datasets [95]. However, many included studies were based on a limited number of patients and samples. More precisely, as shown in Figure 5, the median number of patients in the included studies is 174, which is relatively low for sensitive applications such as polyp detection, bleeding detection, and related tasks.

Another important issue is that almost none of the included studies explicitly reported whether samples were considered and analyzed in a patient-wise manner. In most cases, all images acquired by WCE were pooled, annotated, and used in the training and testing processes. The absence of patient-wise analysis introduces a methodological concern related to data leakage and temporal correlation within WCE data. Since consecutive frames from the same examination are highly similar, random image-level splitting can result in nearly identical samples appearing in both training and testing sets that can lead to artificially inflating performance metrics. This issue is particularly critical in WCE, where thousands of frames originate from a single patient could increase the likelihood of overlap between datasets. As a result, reported accuracy and sensitivity may reflect memorization of patient-specific patterns rather than true generalization. Moreover, this approach does not reflect real-world clinical settings, where personalized models that analyze and make decisions at the individual patient level are crucial for reliable clinical deployment [96].

As seen in Table A1, most included studies showed inconsistent or missing reporting of cohort characteristics, including age, sex, comorbidities, and medication use, with only a limited number of studies providing basic cohort characteristics [34,45]. In 16 studies that provided age data, the median age was 53.5 years, indicating a bias toward older patient populations. These issues reduce the interpretability and clinical relevance of the findings. For example, patients taking blood thinners or antiplatelet drugs may present bleeding patterns that are harder to detect. Without information on medication use, AI models may overlook subtle signs. Likewise, conditions such as liver disease or bleeding disorders can alter lesion appearance and make detection more difficult.

Another technical aspect is the imbalanced data, that influence the training and evaluation of AI models. Multiple studies leveraged the Kvasir dataset [97] for training AI models [47,66,67,81,98,99,100,101]. Despite its popularity, the dataset is significantly imbalanced. For example, while the *Normal Clean Mucosa* category contains over 30,000 images, abnormal classes are drastically underrepresented with *Polyp* with only 55 images, *Blood–Hematin* with 12 images, and *Erythema* with around 159 images. Many available samples in the datasets are also the same abnormality captured at different times, angles, or distances, which reduces the number of truly distinct abnormal cases. This redundancy can cause AI models to overfit to majority classes and perform poorly in detecting rare but clinically important abnormalities.

Moreover, our results reveal a significant under-representation of certain GI regions and conditions. The majority of studies focus on the small bowel, while regions like the esophagus, stomach, and specific segments of the colon receive considerably less attention. Similarly, common conditions such as bleeding and polyps dominate the research landscape, whereas some complex conditions, such as specific inflammatory diseases, are rarely explored. This imbalance restricts the generalizability of AI models.

*Recommendations*: Future research should focus on collecting larger and more diverse datasets that cover a broad range of GI abnormalities, with better representation across lesion types and patient groups. In addition, developing a comprehensive public benchmark dataset, similar to COCO in computer vision [102], would support standardized evaluation and improve the reproducibility of research studies. Moreover, future studies should implement patient-level data collection to have more robust and reliable datasets.

### 5.2. AI Analysis

Although several reviews report promising results, such as sensitivity and specificity values exceeding 90% for the detection of polyps and hemorrhagic lesions [93,94,103], several aspects should be considered when interpreting and drawing conclusions from AI model results. First, our ROB analysis (Figure 8) shows that many of the included studies have unclear or high ROB, particularly in domains related to AI models and methodologies. This indicates that, in most studies, important methodological and AI-related aspects were either not reported or not adequately addressed. Moreover, the observed heterogeneity across studies can be attributed to several factors, including substantial variation in dataset size and composition, differences in AI model architectures, variability in clinical tasks and target pathologies, and inconsistent validation strategies. Included studies ranged from small cohorts to large-scale datasets and employed diverse methodologies such as classification, detection, and segmentation. To further investigate potential sources of this heterogeneity, subgroup analyses were performed based on clinical indication and gastrointestinal location. These stratified analyses enabled a more nuanced assessment of AI performance across different disease categories and anatomical regions, highlighting variations that may account for inconsistencies in pooled estimates and offering more clinically relevant insights into model applicability across diverse settings.

External validation is essential to assess the robustness of AI models in real clinical settings. Without external validation, high performance metrics may not translate reliably to clinical practice.

Several studies report very high performance without external validation. For example, CNN models for detecting obscure GI bleeding have achieved sensitivity and specificity above 98% [56], but their generalizability across different institutions, patient populations, and WCE devices remains uncertain. In contrast, studies that include external validation provide stronger evidence of clinical applicability. Ali et al. [37] conducted a multi-center study on polyp detection and segmentation with external validation and reported a Dice score of 0.82 and accuracy of 98%. Similarly, Xie et al. [33] validated their model across multiple independent centers and achieved a detection rate of 95.9% and sensitivity of 98.8%.

*Recommendation*: We encourage researchers in this domain to apply their methodology in multi-center settings and evaluate the performance of their models on unseen data from completely different cohorts. AI explainability and interpretability techniques can also be used to increase clinicians’ trust in AI models. In addition, given the rapid advances in Large Language Models (LLMs) and their promising performance across various applications [104], it is worth evaluating their potential in this field.

### 5.3. Linking WCE and Conventional Endoscopy

WCE and conventional endoscopy differ substantially in image acquisition. In conventional endoscopy, physicians control illumination, focus, and viewing angle, resulting in more consistent image quality. In contrast, WCE images are acquired passively and often show variations in lighting, resolution, and orientation. As a result, the same lesion may appear differently across the two modalities, which complicates follow-up and confirmation.

This difference limits AI models trained on a single modality. Several domain adaptation approaches have been proposed to address this gap, including Cycle-Consistent Generative Adversarial Networks (CycleGAN), which can translate WCE images to a conventional endoscopy style without paired data [105], and Domain Adversarial Neural Networks (DANN), which aim to learn domain-invariant features [106]. Metric learning approaches may also help align lesions across modalities but require paired datasets, which remain limited [107]. Despite their potential, most of these methods lack validation in real clinical settings, and the scarcity of high-quality paired data remains a key challenge for integrating AI across the full diagnostic workflow.

*Recommendation*: Greater integration between WCE and conventional endoscopy is needed through harmonized annotations and AI models that can operate across both modalities. This would support coherent diagnostic workflows.

### 5.4. Comparison with Expert Interpretation

A key limitation in current AI research on WCE images is the inconsistent comparison between AI performance and that of human experts. Benchmarking AI systems against experienced gastroenterologists is important to assess clinical relevance and added value. Although some studies report improved performance when AI is used as a support tool, such as higher sensitivity in detecting small bowel vascular lesions [54], many studies report only standalone performance metrics. In addition, variability in human interpretation of WCE images has been reported [15], highlighting the need for standardized comparisons to better understand the clinical value of AI systems.

*Recommendation*: Researchers should include direct comparisons with human experts using standardized protocols, and evaluate both diagnostic performance and clinical utility, such as workload and reading time.

### 5.5. Strengths and Limitations

Our review has several strengths. We conducted a comprehensive search across multiple databases using a broad set of keywords, ensuring that we captured a wide range of relevant studies. We also applied QUADAS-2 tool for quality assessment to evaluate the methodological quality and ROB in each study. Additionally, by performing a meta-analysis of the studies, we provide an overview of AI’s diagnostic performance in WCE. However, the main limitation of this review is the high variability among the included studies. Differences in study design, sample sizes, and reporting make it difficult to combine the results of the meta-analysis accurately, which may affect the reliability of its findings. Additionally, this meta-analysis was not prospectively registered in PROSPERO, which may introduce potential risk of reporting bias.

## 6. Conclusions

This systematic review demonstrates that AI has strong potential to improve diagnostic accuracy and efficiency in WCE applications. In contrast to previous reviews that focused on specific diseases, applications, or limited GI regions, our study provides a comprehensive synthesis across multiple AI application domains and GI tract locations, which means it offers a broader and more integrative perspective on the field.

Although many included studies report high sensitivity, specificity, and overall diagnostic accuracy, our analysis identifies several persistent challenges that limit real-world deployment. These include imbalanced datasets, limited patient and sample sizes, incomplete method reporting, ignoring patient-wise model development, and the lack of external and prospective validation in most studies. These observations highlight the need for more uniform methodologies and clinically oriented study designs in future research. Moreover, direct evaluation against experienced gastroenterologists is essential to determine whether AI models provide meaningful added value in real diagnostic workflows.

Furthermore, our meta-analysis confirms the consistently high diagnostic performance of AI models across different clinical indications and GI regions. However, this work emphasizes that methodological rigor, standardized reporting, and robust validation strategies are essential to improve the reliability, generalizability, and clinical adoption of AI-based systems in WCE applications.

## Figures and Tables

**Table 1:** Research questions related to AI applications in WCE.

Question	Description
Q1	Which AI models have been used for WCE across different diagnostic tasks?
Q2	How do AI models perform across studies based on reported performance metrics?
Q3	How do dataset characteristics (size, diversity, imbalance, and annotation quality) influence model performance and generalizability?
Q4	What barriers currently limit the clinical adoption of AI in WCE, and how can future research address these barriers?

**Table 2:** Search criteria of this study.

Group	Search Criteria
G1—AI keywords	Artificial intelligence, Machine learning, Learning algorithms, Deep learning, Unsupervised machine learning, Supervised learning, Image classification, Object detection, Segmentation, Localization
G2—Technical keywords	Capsule endoscopy, Wireless capsule endoscopy, Video capsule endoscopy
G3—Medical keywords	Gastrointestinal, Stomach, Small bowel, Colon, Esophagus, Polyp, Ulcer, Bleeding, Inflammation, Crohn’s disease, Lesion, Celiac, Angiectasias
G4—Document type	English journal articles
G5—Publication year	1 January 2000–1 January 2026
G6—Final result	G1 AND G2 AND G3 AND G4 AND G5

**Table 3:** Inclusion and exclusion criteria of this study.

Inclusion Criteria	Exclusion Criteria
- AI studies on WCE images.	- Studies using traditional statistical models.
- Cohorts comprising relevant patient groups for WCE.	- The primary focus is not AI applications on WCE images.
- Journal articles written in English.	- Non-English and non-journal articles.
	- Review studies.

## Data Availability

This study is a systematic literature review incorporating 72 original research studies published by other scholars. All cited works are listed in the References section. Additionally, we have made all extracted data and relevant information available as Supplementary Files. Further inquiries can be directed to the corresponding author.

## Supplementary Materials

The following supporting information can be downloaded at: https://www.mdpi.com/article/10.3390/diagnostics16091269/s1, This manuscript has six Supplementary Files. Supplementary File S1 presents the search queries. Supplementary File S2 includes the PRISMA checklist and diagram. Supplementary File S3 contains the CHARMS-based data extraction table for all included studies. Supplementary File S4 provides the full risk of bias assessment using the QUADAS-2 tool. Supplementary Files S5 and S6 present the detailed results of the clinical indication-based and location-based meta analyses, respectively.

## Appendix A. Characteristics of Included Studies

**Table A1:** Characteristics of included studies.

First Author	Year	Country	Region of GI Tract	Time Period	Samples	Patients Type	Age	Sex	Data Source	Study Design	Setting
Teng Zhou [86]	2017	China	Small bowel	2008–2009	11 celiac disease patients and 10 controls, analyzed via capsule endoscopy	Patients with celiac disease and controls	50.5 (females mean), 44 (males mean)	45.45% male	Columbia University Medical Center	Retrospective	Clinical practice
Dimitris K. Iakovidis [80]	2018	Greece, UK	Entire GI tract	2015–2018	10,698 images	Patients undergoing endoscopy	-	-	Two public datasets (MICCAI and KID)	Retrospective	Analysis
Zhen Ding [77]	2019	China	Small bowel	2016–2018	6970 patients	Patients with small-bowel diseases and normal variants	-	-	Collected by authors from 77 medical centers	Retrospective	Clinical practice
Romain Leenhardt [30]	2019	France	Small bowel	2011–2018	2946 images with lesions, 600 gastrointestinal angiectasia frames	Patients with gastrointestinal bleeding	72 (mean)	61% male	CAD-CAP database	Retrospective	Analysis
Amit Kumar Kundu [61]	2019	Bangladesh	Entire GI tract	2019	2350 images (450 bleeding, 1900 non-bleeding)	Patients with gastrointestinal abnormalities	-	-	Public dataset	Retrospective	Analysis
Tomonori Aoki [51]	2019	Japan	Small bowel	2009–2018	115 patients (training), 65 patients (validation), 5360 training images, 10,440 validation images	Patients with gastrointestinal abnormalities	63 (training set mean)	54% male (training dataset)	University of Tokyo Hospital	Retrospective	Clinical practice
Sen Wang [31]	2019	China	Entire GI tract	-	1416 videos	Ulcer patients	-	73% male	30 hospitals and 100 medical examination centers, China	Retrospective	Clinical practice
Libin Lan [108]	2019	China	Small bowel	-	7381 wireless capsule endoscopy images	Patients with gastrointestinal abnormalities	-	-	Datasets from Jinshan Science & Technology and Given Imaging	Retrospective	Analysis
U Deding [109]	2020	Denmark	Colon	2016–2018	97 patients	Patients with incomplete optical colonoscopy	64.5 (median)	25 % male	Odense University Hospital (Denmark)	Prospective	Clinical practice
Eyal Klang [45]	2020	Israel	Small bowel	-	17,640 images from 49 patients	Patients with known Crohn’s disease and control subjects	28 (mean)	50% male	Department of Gastroenterology at Sheba Medical Center	Retrospective	Clinical practice
Keita Otani [79]	2020	Japan	Small bowel	2009–2019	167 patients; 398 erosions/ulcers, 538 vascular lesions, 4590 tumors, 34,437 normal images	Patients with gastrointestinal abnormalities	63.6 (mean)	55% male	Tokyo Hospital	Retrospective	Clinical practice
Yixuan Yuan [40]	2020	Hong Kong	Colon	2019	7200 images from 80 patients	Patients with gastrointestinal abnormalities	-	-	Not mentioned	Retrospective	Analysis
Akiyoshi Tsuboi [73]	2020	Japan	Small bowel	-	141 patients (training), 28 patients (validation)	Patients with diagnosed small-bowel angioectasia using capsule endoscopy	68.5 (validation set mean)	54% male (validation set)	Hiroshima University Hospital, University of Tokyo Hospital, Sendai Kousei Hospital	Retrospective	Clinical practice
Tomonori Aoki [60]	2020	Japan	Small bowel	2009–2015	27,847 images (training), 10,208 images (test)	Patients with gastrointestinal abnormalities	53.4 (mean)	56% male	The University of Tokyo Hospital (Japan)	Retrospective	Clinical practice
Hiroaki Saito [32]	2020	Japan	Small bowel	2009–2018	30,584 images for training; 17,507 for testing	Patients with and without small bowel protruding lesions	60.1 (mean)	65.8% male	Sendai Kousei Hospital (Japan), The University of Tokyo (Japan), Hiroshima University Hospital (Japan), Given Imaging (Israel)	Retrospective	Clinical practice
Charles Houdeville [82]	2021	France	Small bowel	2021	1200 frames	Patients with gastrointestinal bleeding	-	-	CAD-CAP	Retrospective	Analysis
Tonmoy Ghosh [69]	2021	USA	Small bowel	-	2350 images (450 bleeding, 1900 non-bleeding)	Patients with suspected intestinal bleeding	-	-	endoscopy.org and KID public datasets	Retrospective	Analysis
Chao Liao [110]	2021	China	Small bowel	-	50,000 frames across 9 videos	Patients undergoing gastrointestinal motility assessment	-	-	Jinshan Science & Technology Company	Retrospective	Clinical practice
Jürgen Herp [111]	2021	Denmark	Colon	-	42 patients, 84 videos	Patients with colonoscopy follow-up need	-	-	Odense University Hospital	Retrospective	Clinical practice
Andrea Caroppo [59]	2021	Italy	Small bowel	-	2352 images (303 with bleeding)	Patients with gastrointestinal bleeding	-	-	Public KID Dataset	Retrospective	Analysis
Furqan Rustam [58]	2021	Pakistan, South Korea	Entire GI tract	-	5000 images from 33 patients	Patients with gastrointestinal tract infections	-	-	Sheikh Zayed Hospital, Rahim Yar Khan	Retrospective	Clinical practice
Ji Xia [68]	2021	China	Stomach	2014–2018	697 patients, 1,023,955 capsule endoscopy images	Patients with gastrointestinal abnormalities	-	-	Changhai Hospital, Shanghai	Retrospective	Clinical practice
Yunseob Hwang [50]	2021	Republic of Korea	Small bowel	2007–2019	526 mall bowel capsule endoscopy videos (training), 5760 independent images (validation)	Patients undergoing mall bowel capsule endoscopy	-	-	Multiple hospitals in Korea	Retrospective	Clinical practice
Miguel José Mascarenhas Saraiva [85]	2021	Portugal	Small bowel	2015–2020	4319 patients, 53,555 capsule endoscopy images	Patients with gastrointestinal abnormalities	-	-	São João University Hospital and ManopH Gastroenterology Clinic	Retrospective	Clinical practice
Tomonori Aoki [112]	2021	Japan	Small bowel	2009–2018	379 patients, 66,028 capsule endoscopy images for training	Patients undergoing small-bowel capsule endoscopy	62.3 (test set mean)	57% male (test set)	University of Tokyo, Hiroshima University Hospital, Sendai Kosei Hospital	Retrospective	Clinical practice
Miguel José Mascarenhas Saraiva [84]	2021	Portugal	Colon	2010–2020	3,387,259 frames from 24 colon capsule endoscopy exams, 3640 images for training/validation	Patients undergoing colon capsule endoscopy for detection of colonic lesions	-	-	São João University Hospital, Porto, Portugal	Retrospective	Clinical practice
Yiftach Barash [44]	2021	Israel	Small bowel	-	17,640 images (7391 ulcers, 10,249 normal)	Patients with Crohn’s disease	-	-	Sheba Medical Center, Tel Hashomer (Israel), Medtronic Dublin (Ireland)	Retrospective	Clinical practice
Miguel Mascarenhas Saraiva [57]	2021	Portugal	Small bowel	2020–2021	6740 images (training and validation)	Patients undergoing DAE for suspected mid-gastrointestinal bleeding	-	-	São João University Hospital, Porto, Portugal	Retrospective	Clinical practice
Eyal Klang [43]	2021	Israel	Small bowel	2009–2018	27,892 images (1942 strictures; 14,266 normal mucosa; 11,684 ulcers)	Crohn’s Disease patients	-	-	Sheba Medical Center, Tel Hashomer (Israel), Medtronic Dublin (Ireland)	Retrospective	Clinical practice
Samir Jain [67]	2021	India, Norway, Czech Republic, Malaysia	Small bowel	2018–2021	Combined KID dataset; images with augmentation	Patients with gastrointestinal anomalies	-	-	KID dataset	Retrospective	Analysis
Miguel Mascarenhas Saraiva [56]	2022	Portugal	Small bowel	2015–2020	1229 patients; 22,095 frames	Patients with suspected gastrointestinal bleeding	-	-	São João University Hospital, Porto, Portugal	Retrospective	Clinical practice
Guillem Pascual [39]	2022	Spain, Denmark	Small intestine, colon	2016–2021	Generic: 1,185,033 frames, Polyp dataset: 248,136 frames, CAD-CAP dataset: 1800 images	Mixed patients	-	-	CAD-CAP, Internal dataset	Retrospective	Analysis
João Afonso [49]	2022	Portugal	Small bowel	2015–2020	1483 wireless capsule endoscopy exams, 6130 frames (4233 containing ulcers and erosions)	Patients with inflammatory bowel disease	-	-	São João University Hospital, Porto, Portugal	Retrospective	Clinical practice
Jun-Xiao Zhou [113]	2022	China	Small bowel	2016–2019	277 polyp images from 480 patients	Patients with gastrointestinal disorders	-	-	Guangzhou First People’s Hospital, CVC-Colon, CVC-Clinic	Retrospective	Clinical practice
Md. Jahin Alam [66]	2022	Bangladesh, USA	Entire GI tract	2016–2018	47,238 images	Patients with gastrointestinal abnormalities	-	-	Kvasir-Capsule public dataset	Retrospective	Analysis
Naoki Higuchi [42]	2022	Japan	Colon	2018–2020	739,021 images	Patients with moderate or mild ulcerative colitis	-	-	Hirosaki University Hospital, Japan	Prospective	Clinical practice
João Pedro Sousa Ferreira [48]	2022	Portugal	Small bowel and Colon	2017–2020	8085 images	Patients with Crohn’s Disease	-	-	São João University Hospital and ManopH Gastroenterology Clinic, Portugal	Retrospective	Clinical practice
Saqib Mahmood [47]	2022	Pakistan	Entire GI tract	2016–2018	47,238 labeled frames from 117 videos	Patients with GI tract abnormalities	-	-	Kvasir-Capsule dataset	Retrospective	Analysis
Meryem Souaidi [114]	2022	Morocco	Entire GI tract	-	WCE, PillCam-COLON, CVC-ClinicDB, ETIS-Larib, PASCAL VOC, COCO	Patients with gastrointestinal polyp indications	-	-	PillCam-COLON, CVC-ClinicDB, ETIS-Larib, PASCAL VOC, COCO	Retrospective	Analysis
Miguel Mascarenhas [38]	2022	Portugal	Colon	2010–2020	124 patients, 5715 colon capsule endoscopy frames	Patients with protruding lesions detected via colon capsule endoscopy	-	-	São João University Hospital and ManopH Gastroenterology Clinic	Retrospective	Clinical practice
Tom Kratter [46]	2022	Israel	Small bowel, Colon	-	33,100 capsule endoscopy images	Patients with Crohn’s disease and normal mucosa	-	-	Sheba Medical Center, Tel Hashomer (Israel), Medtronic Dublin (Ireland)	Retrospective	Clinical practice
Xia Xie [33]	2022	China	Small bowel	2012–2021	5825 small bowel capsule endoscopy examinations	Patients undergoing small bowel capsule endoscopy	49.8 (mean)	60.9% male	51 medical centers, China	Retrospective	Clinical practice
SangYup Oh [75]	2023	South Korea	Small bowel	2002–2022	1,431,344 images (across 260 cases)	Mixed gastrointestinal patients	-	-	Dongguk University Ilsan Hospital, South Korea	Retrospective	Clinical practice
Raizy Kellerman [41]	2023	Israel	Small bowel	2011–2021	101 patients	Newly diagnosed Crohn’s patients	27 (median)	46.5% male	Sheba Medical Center, Israel	Retrospective	Clinical practice
Mehrdokht Bordbar [34]	2023	Iran	Entire GI tract	-	29 patients, 14,691 frames	Mixed gastrointestinal patients	53.5 (mean)	66% male	Namazi Hospital, Shiraz, Iran	Retrospective	Clinical practice
Sharib Ali [37]	2023	UK, Norway, France, Italy, Egypt	Colon	-	300 patients, 8037 frames	Patients undergoing colonoscopy for colorectal cancer screening	-	-	Public PolypGen dataset	Retrospective	Analysis
Ye Chu [55]	2023	China	Small bowel	2014–2020	12,403 images	Patients with angiodysplasias undergoing capsule endoscopy	-	-	Ruijin Hospital, China	Retrospective	Clinical practice
Sofia A. Athanasiou [72]	2023	Greece	Entire GI tract	2019–2020	6016 frames	Patients with bleeding, angiodysplasias, hemangiomas, or lesions predisposing to bleeding	-	-	Kapodistrian University of Athens, Attikon University Hospital, Laikon University Hospital, Aristotle University of Thessaloniki	Prospective	Clinical practice
Akihiko Sumioka [115]	2023	Japan	Small bowel	2011–2021	26 patients	Primary small-bowel follicular lymphoma patients	60.9 (mean)	50% male	Hiroshima University Hospital, Japan	Retrospective	Clinical practice
Javeria Naz [65]	2023	Pakistan, Norway, UK, Saudi Arabia	Entire GI tract	2016–2018	>30,000 images	Patients with gastrointestinal abnormalities	-	-	Kvasir-V1 and other pulic sources	Retrospective	Analysis
Ayako Nakada [74]	2023	Japan	Small bowel	2009–2019	651 patients, >10 million wireless capsule endoscopy images	Patients with obscure gastrointestinal bleeding, small intestine tumors, abdominal symptoms	-	-	Nine hospitals across Japan	Retrospective	Analysis
Tomonori Aoki [116]	2024	Japan	Small bowel	2009–2019	36 wireless capsule endoscopy videos, 43 lesions	Patients with abnormal lesions in wireless capsule endoscopy	-	-	University of Tokyo, Hiroshima University Hospital, Sendai Kosei Hospital, and Japan Medical Association	Retrospective	Clinical practice
Ali Sahafi [22]	2024	Denmark, Poland	Colon	-	2500 images	Patients with gastrointestinal abnormalities	-	-	KID Dataset	Retrospective	Analysis
Rui-Ya Zhang [54]	2024	China	Small bowel	2013–2023	111,861 images	Patients with gastrointestinal issues	-	-	Shanxi Provincial Hospital, China	Retrospective	Clinical practice
Tsedeke Temesgen Habe [81]	2024	Finland	Entire GI tract	2016–2018	47,238 images (class-annotated)	Patients with gastrointestinal abnormalities	-	-	Kvasir-Capsule public dataset	Retrospective	Analysis
Mohamed Achraf Belabbes [100]	2024	Morocco	Entire GI tract	2023–2024	Several datasets: PillCam COLON, Kvasir-SEG, ETIS-Larib, CVC-ClinicDB	Patients with suspected polyps	-	-	PillCam COLON, Kvasir-SEG, ETIS-Larib, CVC-ClinicDB	Retrospective	Analysis
Kyung Seok Choi [53]	2024	South Korea	Small bowel	2018–2020	103 paitient (1,037,286 images)	Patients with suspected small-bowel bleeding (SSBB)	64.7 (mean)	62.2% male	Consecutive patient data from Yeouido St. Mary’s and Seoul St. Mary’s Hospitals	Retrospective	Clinical practice
Akihito Yokote [70]	2024	Japan	Small bowel	2014–2021	954 patients, 18,481 images	Patients undergoing small bowel capsule endoscopy	-	-	Kyushu University Hospital	Retrospective	Clinical practice
Dong Liu [98]	2024	China	Colon	2016–2018	3 public datasets: 1000 polyp images (Kvasir-SEG), 590 images (Kvasir-Instrument), 55 images (KvasirCapsule-SEG)	Patients with gastrointestinal abnormalities	-	-	Kvasir-Capsule public dataset	Retrospective	Analysis
Xia Xie [76]	2024	China	Stomach and Small bowel	2021–2022	1069 training, 342 validation	Patients with gastrointestinal abnormalities	42 (median)	45.61% male	Three hospitals in Chongqing (China), University of Southern Denmark, Jinshan Science & Technology (China)	Retrospective	Clinical practice
Seung-Joo Nam [83]	2024	Republic of Korea	Stomach, Small bowel, Colon	2018–2021	126 paitient (2,392,462 images)	Patients with gastrointestinal abnormalities	-	-	Kangwon National University Hospital (South Korea), Dongguk University Ilsan Hospital (South Korea)	Retrospective	Clinical practice
Lan Li [64]	2024	China	Small bowel	2016–2022	1452 patients for training; 298 for testing	Patients with small bowel lesions	-	-	Zhejiang University	Retrospective	Clinical practice
Esmaeil S. Nadimi [36]	2024	Denmark	Large Intestine (Colon)	2021	5838 polyp & 5573 normal images (augmented) for recognition; 5838 for size; 144 (49 neoplastic, 95 non-neoplastic) for characterization	FIT-positive screening participants undergoing colon capsule endoscopy	-	-	Odense University Hospital and University of Southern Denmark	Retrospective	Analysis
Yeong Seok Kwon [117]	2025	Korea	Small Bowel	2011–2022	28,279 small-bowel images, 32 full-length SBCE videos for external validation	Patients undergoing small-bowel capsule endoscopy for suspected bleeding	-	-	Chuncheon & Dongtan Sacred Heart (training); Yeungnam & Ewha Univ. Hospitals (external)	Prospective	Clinical validation
Miguel José Mascarenhas Saraiva [35]	2025	Portugal	Small and Large Intestine	2021–2023	191,455 frames extracted from 1245 CE/CCE exams	Patients undergoing capsule or colon capsule endoscopy for diagnostic evaluation	-	-	CHU São João & ManopH Clinic (Portugal)	Retrospective	Analysis
Patrícia Andrade [118]	2025	Multiple (Europe & USA)	Small Intestine	2021–2024	259 SBCE exams (PillCam SB3 and Olympus EC-10)	Crohn’s disease patients undergoing small-bowel capsule endoscopy	-	-	Multiple hospitals across Europe and the USA	Retrospective	Clinical practice
Miguel Mascarenhas Saraiva [119]	2025	Portugal, Spain, Brazil, USA	Entire GI tract	-	330 capsule endoscopy videos	Patients undergoing capsule endoscopy across Portugal, Spain, Brazil and the USA	-	-	Multi-centre dataset using PillCam, Olympus and OMOM systems	Prospective	Clinical practice
Maxime Le Floch [78]	2025	Germany	Small Intestine	2011–2023	80 VCE recordings; 3,513,539 frames annotated	Patients undergoing video capsule endoscopy	-	-	University Hospital Carl Gustav Carus, Germany	Retrospective	Analysis
Miguel Martins [63]	2025	Portugal, Spain	Esophagus and Stomach	2021–2023	59,482 frames extracted from 774 capsule endoscopy procedures	Patients undergoing capsule endoscopy of the oesophagus and stomach	-	-	São João University Hospital, Portugal & La Princesa University Hospitals, Spain	Retrospective	Analysis
Charles Houdeville [52]	2025	France, Netherlands	Small Intestine	2019–2021	148 patients; 1525 images	Patients with suspected small-bowel bleeding undergoing SBCE	Training: 72 (mean); Validation: 78 (mean)	52 % male	Sorbonne University Hospital, Paris & Radboud University Medical Center, Nijmegen	Retrospective	Analysis
Jian Chen [62]	2025	China	Small Intestine	-	34,799 images from multiple public datasets	Patients undergoing small-bowel capsule endoscopy with various lesions	-	-	Multi institutional datasets from Chinese hospitals and public sources	Retrospective	Analysis
Bernardo Rosa [71]	2025	Portugal	Entire GI tract	2024	100 patients	Patients undergoing panenteric capsule endoscopy for suspected mid-to-lower GI bleeding	66.5 (median)	35 % male	Hospital da Senhora da Oliveira & São João University Hospital, Portugal	Prospective	Clinical practice

**Table A2:** AI-related characteristics of included studies.

First Author	Gastrointestinal Problem	Application	AI Models	Performance Metrics	Cross Validation	External Validation
Teng Zhou [86]	Detection of celiac disease	Image classification	GoogLeNet	Acc: 100%, Sens: 100%, Spec: 100%	7-fold cross-validation performed	No
Dimitris K. Iakovidis [80]	Detecting and localizing gastrointestinal anomalies	Image classification and anomaly localization	Weakly supervised CNN with saliency detection	Classification AUC (mean): 88.85%, localization (mean): AUC: 0.86	10-fold cross-validation	Yes
Zhen Ding [77]	Detection of inflammation, ulcer, polyps, lymphangiectasia, bleeding, vascular disease, protruding lesion	Image classification	ResNet	Per-patient (Sens: 99.88%, NPV: 99.77%), Per-lesion (Sens: 99.90%, NPV: 99.77%)	No	No
Romain Leenhardt [30]	Detection of GI angiectasia	Image classification and segmentation	CNN	Sens: 100%, Spec: 96%, Prec: 96.15%, NPV: 100%	No	No
Amit Kumar Kundu [61]	Detection and localization of bleeding regions	Image classification and bleeding zone segmentation	Support vector machine	Acc: 96.77%, Sens: 97.55%, Spec: 96.59%	10-fold cross-validation	No
Tomonori Aoki [51]	Detection of erosions and ulcerations in the small bowel	Image classification	SSD	Acc: 90.8%, Sens: 88.2%, Spec: 90.9%, AUC: 0.958	No	No
Sen Wang [31]	Ulcers detection	Image classification	ResNet34	Acc: 92.05%, Sens: 91.64%, Spec: 92.42%, prec: 92.37%, F1 score 91.99%, AUC: 0.97	5-fold cross-validation	No
Libin Lan [108]	Detection of bleeding, polyps, and tumors	Image classification	CascadeProposal (based on Fast R-CNN)	Performance average: overall mAP: 72.17%, per-Class mAP: 70.54%, region proposal methods mAP: 69.40%	No	No
U Deding [109]	Polyp detection	Image localization and tracking	AI-based tracking algorithm	Acc (mean): 77%	No	No
Eyal Klang [45]	Ulcers detection in Crohn’s disease	Image classification	Xception	Acc: 96%, Sens: 94.8%, Spec: 97%, Prec: 95.8%, NPV: 96.4%, AUC: 0.99%	5-fold cross-validation	No
Keita Otani [79]	Detection of small bowel lesions	Image classification and object detection	RetinaNet and SSD	RetinaNet (best model) performance average: AUC: 0.93	5-fold cross-validation	Yes
Yixuan Yuan [40]	Polyp detection	Image classification	DenseNet-UDCS	Acc: 93.19%, Sens: 95.12%, Prec: 94.56%, F1 score: 94.84%	10-fold cross-validation	No
Akiyoshi Tsuboi [73]	Detection of small-bowel angioectasia	Image classification	SSD	Sens: 98.8%, Spec: 98.4%, AUC: 0.99	No	Yes
Tomonori Aoki [60]	Blood content detection	Image classification	ResNet50	Acc: 99.89%, Sens: 96.63%, Spec: 99.96%, Prec: 98.06%, NPV: 99.93%, AUC: 0.99, Sens: 96.63%	No	No
Hiroaki Saito [32]	Detection of polyps, nodules, epithelial tumors, submucosal tumors, and venous structures	Image classification	SSD	Sens: 90.7%, Spec: 79.8%, AUC: 0.91	No	No
Charles Houdeville [82]	Angiectasias detection	Image classification	Axaro	Performance average: Acc: 99.7%, Prec: 98.2%, NPV: 96.9%	No	Yes
Tonmoy Ghosh [69]	Detection of bleeding	Image classification, bleeding zone segmentation	AlexNet, SegNet	Bleeding classification (F1 score: 98.49%, Sens: 97.51%, Spec: 99.88%, Prec: 99.50%, Acc: 99.44	No	No
Chao Liao [110]	GI motility assessment	Region-based in consecutive WCE frames	ResNet-50)	Percentage of correct key-points: 66.49%	No	No
Jürgen Herp [111]	Screening for polyps, inflammatory diseases, and tumors	Capsule localization and path reconstruction	Feature Point Tracking with filtering	Path difference: 4 ± 0.7 cm, Classification Acc: 86%, Section Acc: 92%	No	Yes
Andrea Caroppo [59]	Bleeding and lesion classification	Image classification	Fusion of VGG19, InceptionV3, and ResNet50	Performance average: Acc: 96.95%, Sens: 97.26%, Spec: 96.41%, Prec: 97.79%, F1 score: 97.52%	10-fold cross-validation	No
Furqan Rustam [58]	Bleeding detection	Image classification	MobileNet+custom CNN	Acc: 99%, Prec: 100%, Recall: 99.4%, F1 score: 99.7%	10-fold cross-validation	No
Ji Xia [68]	Detection of various gastric lesions	Image classification	ResNet-34 and Faster-RCNN	Performance average: Acc: 81.17%, Sens: 94.89%, Spec: 68.77%, Prec: 55.57%, NPV: 99.90%, AUC: 0.84	No	No
Yunseob Hwang [50]	Classification and localization of small-bowel lesions	Image classification and lesion localization	VGGNet	Performance average: Acc: 96.73%, Sens: 96.34%, Spec: 96.04%, Prec: 96.11%, NPV: 96.41%, AUC: 0.99	10-fold cross-validation	Yes
Miguel José Mascarenhas Saraiva [85]	Detection small bowel lesions	Image classification	Xception	Acc: 98%, Sens: 87.8%, Spec: 99.4%, NPV: 99.4%	No	No
Tomonori Aoki [112]	Detection of mucosal breaks, angioectasia, Protruding lesions, and bleeding	Image classification	SSD and ResNet50	Performance average: Acc: 92.60%, Sens: 99.36%, Spec: 81.18%	No	No
Miguel José Mascarenhas Saraiva [84]	Protruding polyps, epithelial tumors, submucosal tumors, and nodes	Image classification	Xception	Acc: 92.2%, Sens: 90.7%, Spec: 92.6%, Prec: 79.2%, NPV: 96.9%, AUC: 0.97	No	No
Yiftach Barash [44]	Ulcer severity in Crohn’s disease	Image classification and ulcer severity grading	Ordinal CNN (ResNet based)	Performance average: Acc: 77.13%, Sens: 66.20%, Spec: 84.57%, F1 score: 67.5%, AUC: 0.82	5-fold cross-validation	No
Miguel Mascarenhas Saraiva [57]	Detection of GI angioectasia	Image classification	Xception	Acc: 95.3%, Sens: 88.5%, Spec: 97.1%, Prec: 88.8%, NPV: 97%, AUC: 0.98,	No	No
Eyal Klang [43]	Detection of intestinal strictures in Crohn’s Disease	Image classifcation	EfficientNetB5	Performance average: Acc: 86.2%, Sens: 92%, Spec: 89%, Prec: 55%, NPV: 99%, F1 score: 69%, AUC: 0.93	10-fold cross-validation	No
Samir Jain [67]	Localization and detection of inflammatory, polyp, vascular, and normal mucosa	Image classification	WCENet (Custom CNN, Grad-CAM++, SegNet)	Performance average: Acc: 98%, Sens: 98%, Spec: 98%, Prec: 98%, F1 score: 0.98, AUC: 0.99, localization IoU: 0.60	5-fold cross-validation	No
Miguel Mascarenhas Saraiva [56]	Obscure gastrointestinal bleeding	Image classification	Xception	Sens: 98.6%, Spec: 98.9%, Acc: 98.5%, Prec: 98.7%, AUC: 1.0	No	No
Guillem Pascual [39]	Polyp detection	Image classification	ResNet with SimCLR, TLBA	Acc: 92.77%, AUC: 0.95	5-fold cross-validation	No
João Afonso [49]	Detection of ulcers and erosions	Image classification with bleeding risk	Xception	Acc: 95.6%, Sens: 90.8%, Spec: 97.1%, Prec: 93.4%, NPV: 97.1%	No	No
Jun-Xiao Zhou [113]	Polyp segmentation	Image segmentation	Ensemble with SegNet, U-Net, Attention-UNet, ResNet-UNet, HarDMSEG	Guangzhou First People’s Hospital dataset (IoU: 0.55, Dice: 0.65), CVC datasets (IoU: 0.84, Dice: 0.90)	No	No
Md. Jahin Alam [66]	Detection of ulcer, erosion, bleeding, lymphangiectasia, and angiectasia	Image classification	RAt-CapsNet	Binary (Acc: 98.51%, Prec: 99.02%, Sens: 98.45%, F1 score: 98.73%), 3-class Acc: 97.92%, 4-class: 95.65%	No	No
Naoki Higuchi [42]	Ulcerative colitis	Image classification for severity scoring	ResNet50	Acc: 97.3%	No	No
João Pedro Sousa Ferreira [48]	Detection of ulcers and erosions in Crohn’s disease	Image classification	Xception	Acc: 92.4%, Sens: 90.0%, Spec: 96.0%, Prec: 96.6%, NPV: 99.5%, AUC: 1	No	No
Saqib Mahmood [47]	Detection of peptic Ulcer and other GI disorders	Image classification	GI Disease-Detection Network with BL-SMOTE	Acc: 98.9%, Prec: 98.9%, Sens: 98.8% AUC: 0.99, F1 score: 98.9%	No	No
Meryem Souaidi [114]	Polyp detection	Object detection	MP-FSSD	Average mAP: 93.4%	5-fold cross-validation	Yes
Miguel Mascarenhas [38]	Protruding polyps, epithelial tumors, and subepithelial lesions	Image classification	Xception	Acc: 95.3%, Sens: 90.0%, Spec: 99.1%, AUC: 0.99	3-fold cross-validation	No
Tom Kratter [46]	Detection and grading of ulcer	Image classification and grading	EfficientNetB4	Performance average: Acc: 87.87%, F1 score: 97.8%, AUC: 0.97	5-fold cross-validation	No
Xia Xie [33]	Detection of small bowel abnormalities	Image classification	EfficientNet and YOLO	Sens: 93.45%	No	Yes
SangYup Oh [75]	Detection of small bowel lesions	Video classification	Transformer-based VWCE-Net	Sens: 95.1%, Spec: 83.4%	No	No
Raizy Kellerman [41]	Predicting the need for biological therapy in Crohn’s Disease	Video classification	TimeSformer	Acc: 81%, Prec: 81%, Sens: 75%, Spec: 84% AUC: 0.86%	5-fold cross-validation	No
Mehrdokht Bordbar [34]	Detection of ulcers, bleeding, polyps, and erosions, and vascular abnormalities	Image classification	3D-CNN	Acc: 99.20%, Sens: 98.92%, Prec: 99.51%, NPV: 98.92%, F1 score: 99.21%	No	Yes
Sharib Ali [37]	Polyp detection and segmentation	Image classification and segmentation	FCN-8s, U-Net, PSPNet, DeepLabV3+, ResNet-UNet	Single frame: (Acc: 98%, Dice score: 0.82, Prec: 92%, Sens: 81%), Sequence-based (Acc: 97%, Dice score: 0.71, Prec: 90%, Sens: 73	No	Yes
Ye Chu [55]	Segmentation angiodysplasias	Image segmentation	ResNet50 with feature fusion	Acc: 99%, mIOU: 0.69, PPV: 94.27%, NPV: 98.74%	No	No
Sofia A. Athanasiou [72]	Organ boundary detection	Image classification	CNN	Acc: 95.56%, Sens: 91.82%,	No	Yes
Akihiko Sumioka [115]	Disease surveillance evaluation of primary small-bowel follicular lymphoma	Image classification and disease surveillance	EfficientDet	Acc: 85.6%, Sens: 81.2%, Spec: 88.6%, AUC: 0.91	No	No
Javeria Naz [65]	Detection of GI abnormalities	Image classification	XcepNet23 and ResNet18	Average performance (Acc: 99.62%, Sens: 99.34%, Spec: 99.94%, Prec: 99.34%, F1 score: 100%)	5-fold cross-validation	No
Ayako Nakada [74]	Detection of ulcerations, vascular lesions, and tumors	Object detection	Revised RetinaNet	Performance average: Acc: 93.87%, Sens: 89.10%, Spec: 94.73%, IoU: 82.33%, AUC: 0.99	5-fold cross-validation	No
Tomonori Aoki [116]	Detection of mucosal breaks, angioectasia, protruding lesions	Image classification	SSD, EfficientDet	SSD Sens: 67%, EfficientDet Sens: 79%	No	No
Ali Sahafi [22]	Polyps segmentation	Image segmentation	YOLO-V8	Prec: 98%, Recall: 97.9%, mAP: 97.4%, Dice score: 97.94%	No	No
Rui-Ya Zhang [54]	Various small bowel lesions with bleeding risks	Image classification and lesion detection	ResNet-50 and YOLO-V5	Acc: 98.96%, Sens: 99.17%, Spec: 99.92%, AUC: 0.98	No	No
Tsedeke Temesgen Habe [81]	Detection of polyps, angiectasia, erosions, ulcers, and bleeding	Object detection and classification	SSD300, EfficientDet, Faster R-CNN, RetinaNet, YOLOv3, RTMDet, Faster R-CNN+ResNet	Best performance: Prec: 99.67%, Sens: 99.67%, Spec: 99.97%, F1 score: 99.67%	5-fold cross-validation	No
Mohamed Achraf Belabbes [100]	Polyp detection	Object detection and localization	SPDNet (SSD based)	Performance average: mAP: 91.7%	5-fold cross-validation	Yes
Kyung Seok Choi [53]	Detection of small-bowel lesions	Image classification	VGGNet	Acc: 96%, Sens: 96.5%, Spec: 95.8%, AUC: 0.99	No	No
Akihito Yokote [70]	Detection of small-bowel lesions	Image classification and object detection	YOLOv5	Sens: 91%, Prec: 67.6%. F1 score: 77.6%, AUC: 0.98	3-fold cross-validation	No
Dong Liu [98]	Segmentation of colonic polyps	Image segmentation	NA-SegFormer	Performance average: Dice Score: 90.54%, Acc: 93.04%, IoU: 85.71%, Prec: 86.84%, Recall: 95.64%	5-fold cross-validation	Yes
Xia Xie [76]	Detection of gastric and small bowel lesions	Image classification	EfficientNet and YOLO	Performance average: Acc: 96.93%, Sens: 97.77%, Spec: 99.80%	No	No
Seung-Joo Nam [83]	Real-time organ localization and transit time estimation	Localization and transit time	Hybrid CNN	Average classification performance (Acc: 97.1%, F1 score: 97.1%, Sens: 96.87%, Spec: 98.33%), transit time performance (gastric transit time error: 4.3 ± 9.7 min, small bowel transit time error: 24.7 ± 33.8 min )	No	No
Lan Li [64]	Detection of small-bowel lesions	Image classification and localization	YOLOv5	Performance average: Acc: 92.76%, Sens: 93.86%, Spec: 91.54%	No	Yes
Esmaeil S. Nadimi [36]	Colonic polyp detection, size estimation and neoplastic classification	Recognition and characterisation	NasNetLarge-based recognition; VGG16-based classifier	Recognition: Sensitivity 99.9%, Specificity 99.4%, NPV 99.8%; Characterisation: Sensitivity 82%, Specificity 80%, Accuracy 81%	No	No
Yeong Seok Kwon [117]	Obscure gastrointestinal bleeding (erosions/ulcers, angiodysplasia, bleeding)	Lesion classification	DenseNet201 ensemble with post filter	Accuracy 99.4% (erosions/ulcers), 99.8% (angiodysplasia), 99.9% (bleeding); Sensitivity 90.1% (erosions/ulcers), 100% (angiodysplasia & bleeding); Specificity 99.7%, 99.8%, 99.9%	No	Yes
Miguel Jose Mascarenhas Saraiva [35]	Protruding lesions (polyps, epithelial tumours, subepithelial lesions)	Image classification	Modified ResNet	Sensitivity: 79.7%, Specificity 96.5%, PPV 81.5%, NPV 96.0%, Accuracy 93.7%	Yes	No
Patricia Andrade [118]	Ulcers and erosions in Crohn’s disease	Detection	Convolutional neural network	AI review: Sensitivity 90.2%, Specificity 84.4%, PPV 76.1%, NPV 94.0%, Accuracy 86.5%	No	Yes
Miguel Mascarenhas Saraiva [119]	Pleomorphic lesions across entire GI tract	Detection	Deep neural network	Sensitivity 97.5%, Detection rate 96.1% (605/635 lesions)	No	Yes
Maxime Le Floch [78]	Multi-label classification of technical artefacts, view quality, anatomical segments and pathologies	Multi-task classification	ResNet-50	Anatomical segments: Micro F1 0.89, Macro F1 0.71, Macro accuracy 0.81, Micro accuracy 0.89	Yes	No
Miguel Martins [63]	Pleomorphic oesophageal and gastric lesions	Detection	CNN with cross-covariance transformer (XCiT)	Test set: Sensitivity 92.2%, Specificity 95.1%, PPV 79.3%, NPV 98.3%, Accuracy 94.6%	Yes	No
Charles Houdeville [52]	Small bowel capsule endoscopy lesion detection	Classification	Random forest	Specificity 91.1%, AUC 0.873, Accuracy 84.2%	No	Yes
Jian Chen [62]	Multiple small bowel lesions	Multi-task classification	FocalNet (transformer-based)	Precision 88.12%, Sensitivity 85.69%, F1 85.84%, Specificity 98.58%, Accuracy 85.69%, AUC 0.98	No	Yes
Bernardo Rosa [71]	Potential haemorrhagic lesions across entire GI tract	Detection	AI PCE method	Sensitivity 95.2%, Specificity 97.3%, PPV 98.4%, NPV 92.3%, AUROC 0.963	No	Yes

Acc: Accuracy, AUC: Area Under the Curve, ANN: Artificial Neural Network, BL-SMOTE: Borderline Synthetic Minority Over-sampling Technique, CE: Capsule Endoscopy, CNN: Convolutional Neural Network, COCO: Common Objects in Context, GI: Gastrointestinal, IoU: Intersection over Union, mAP: Mean Average Precision, mIOU: Mean Intersection over Union, MAE: Mean Absolute Error, NPV: Negative Predictive Value, PASCAL VOC: Visual Object Classes, PPV: Positive Predictive Value, Pr: Precision, Sens: Sensitivity, SimCLR: Simple Framework for Contrastive Learning of Visual Representations, SGD: Stochastic Gradient Descent, SPDNet: Symmetric Positive Definite Network, Spec: Specificity, SSD: Single Shot Multibox Detector, TLBA: Triplet Loss-Based Approach, WCE: Wireless Capsule Endoscopy.
