# Supplementary material for: Artificial Intelligence in Gastrointestinal Wireless Capsule Endoscopy: A Systematic Literature Review and Meta-Analysis
Source: Diagnostics (Basel). 2026 Apr 23;16(9):1269. doi: 10.3390/diagnostics16091269 (PMC13162847; doi:10.3390/diagnostics16091269)
Supplement: Supplementary file 1 [file diagnostics-16-01269-s001.zip › S1_search_queries.pdf]

PubMed Search Query:

((((((Artificial intelligence) OR (Machine learning) OR (Deep learning) OR (Unsupervised machine learning) OR (Supervised learning) OR(Image classification) OR (Object detection) OR (Segmentation) OR (Localization))) AND (Capsule endoscopy) OR (Video capsule endoscopy)) AND ((Gastrointestinal) OR (Stomach) OR (Small bowel) OR (Colon) OR (Esophagus) OR (Polyp) OR (Ulcer) OR (Bleeding) OR (Inflammation) OR (Crohn's disease) OR (Lesion) OR (Celiac) OR (Angiectasias)) AND(English[Language])) AND (("2000/01/01"[Date - Publication] : "2025"[Date - Publication]))) NOT ((systematic review[Publication Type]) OR (Meta-Analysis[Publication Type]))

Web of Science Search Query:

(TS=(artificial intelligence) OR TS=(machine learning) OR TS=(deep learning)) AND ALL=(capsule endoscopy) AND LA=(English) AND PY=(2000-2025) AND DT=(Article)

Scopus and Google Scholar Search terms:

("artificial intelligence" OR "machine learning" OR "deep learning") (Topic) and "capsule endoscopy" (All Fields) and English (Language) and 2000-2025 (Year Published)

MeSH Terms:

"artificial intelligence", machine intelligence, learning machine, information processing, artificial neural network, "machine learning", "deep learning", "capsule endoscopy", "wireless capsule endoscopy", "inflammatory bowel diseases", "gastrointestinal haemorrhage" , "small intestine gastrointestinals", "gastrointestine tract", "large intestine", "colonic polyp", "Crohn's disease", "colonic ulcer", "stomach ulcer".
